# The symbiotic relationship between educational robotics and computer science in formal education

**DOI:** 10.1007/s10639-021-10494-3

**Published:** 2021-04-04

**Authors:** Laila El-Hamamsy, Barbara Bruno, Frédérique Chessel-Lazzarotto, Morgane Chevalier, Didier Roy, Jessica Dehler Zufferey, Francesco Mondada

**Affiliations:** 1 MOBOTS Group, EPFL, Lausanne, Switzerland; 2 LEARN – Center for Learning Sciences, Ecole Polytechnique Fédérale de Lausanne (EPFL), Lausanne, Switzerland; 3 Computer-Human Interaction In Learning and Instruction (CHILI) Laboratory, EPFL, Lausanne, Switzerland; 4 University of Teacher Education (Haute Ecole Pédagogique) Vaud, Lausanne, Switzerland; 5 Flowers Team, INRIA, Université de Bordeaux, Ensta Paris Tech, Bordeaux, France

**Keywords:** Educational robotics, Computer science education, Formal learning, Teacher professional development, Elementary education

## Abstract

Educational Robotics (ER) has the potential to provide significant benefits to education, provided an increase in outreach by transitioning from the extra-curricular initiatives in which ER has thrived to formal education. As Computer Science (CS) Education is undergoing curricular reforms worldwide, the present study addresses the case of a Digital Education reform that included ER as a means to teach core CS concepts. Approximately 350 teachers from the first four grades of primary school participated in a mandatory two-year continuing professional development (CPD) program. The first year of the program was dedicated to CS and introduced teachers to CS Unplugged (CSU) and Robotics Unplugged (RU) activities. As such, we analyse the interplay between these activities and focus on teachers’ voluntary adoption of the proposed content in classrooms. This is complemented by an analysis of their perception and recommendation of ER. The findings highlight three main points. Firstly, ER benefits from the integration in the CS CPD, as this provides the necessary traction to introduce ER into teacher practices (the teachers freely devoted 2275 h to ER activities in their classrooms, over two years). Secondly, the presence of ER activities in the CS-CPD allows a higher proportion of teachers to adopt the CS content, as there are teachers that favour one type of activity over the other. Finally, the globally positive perception of ER registered in this study is relevant for two reasons: teachers were not voluntarily participating in the CPD, and results did not differ between pioneers and novices.

## Introduction

Educational Robotics (ER) envisions the use of robots as a tool to enhance teaching (Hamner et al., 2016) and learning (Miller & Nourbakhsh, 2016). A large number of studies have investigated the benefits of conducting ER activities: in addition to helping achieve the desired learning outcomes, they improve students’ motivation (Daniela & Lytras, 2019; Greca Dufranc et al., 2020) and support inclusive education (Daniela & Lytras, 2019); e.g. for students with special needs (Kim et al., 2015), different socio-economic status or culture (Eguchi, 2015) and / or gender (Sullivan & Bers, 2019). Although these advantages are perceived by both researchers and teachers (Chevalier et al., 2016; Castro et al., 2018; Khanlari, 2019; Negrini, 2019, 2020), the struggle to integrate ER into formal education is well documented (Eguchi, 2014; Chevalier et al., 2016; Benitti & Spolaôr, 2017; Negrini, 2020). The reasons for this difficulty are commonly identified in a number of first order (i.e. external) and second order (i.e. internal) barriers to technology integration (Ertmer, 2005). From this perspective, the introduction of ER as an extra-curricular activity in informal learning environments can be seen as an attempt to circumvent these barriers (Benitti & Spolaôr, 2017; Greca Dufranc et al., 2020). While the use of robotics in informal contexts has helped explore the range of possibilities ER has to offer, this comes at the price of limited outreach, since it relies on having both flexibility in the curriculum and innovative teachers and pioneers in the matter. A change in scale via curricular reform is thus required as confining ER to informal education not only limits its accessibility, but also increases the gap between teachers and students who do not require additional incentive to engage in ER activities, and those who do. Curricular reform would also help address recurrent first order barriers, such as cost, limited access to resources, lack of time and adequately trained teachers (Kradolfer et al., 2014; Chevalier et al., 2016; Mondada et al., 2017; Castro et al., 2018; Negrini, 2020). Unfortunately, ER-related curricular reform still seems to be a distant reality in most countries.

Conversely, efforts to integrate Computer Science (CS), to which ER is increasingly associated, into formal education are numerous (Thompson et al., 2013; Heintz et al., 2016; The Royal Society, 2017; The Committee on European Computing Education (CECE) (2017); Webb et al., 2017) and, albeit not exempt from difficulties (Heintz et al., 2016; Webb et al., 2017; The Royal Society, 2017; Roche, 2019), they are often better documented, and dare we say, more successful. However, it is unclear how and to what extent Educational Robotics is part of those endeavours and is adopted by teachers into their practices (Balanskat & Engelhardt, 2015; Fraillon et al., 2020; European Union & Education, 2019).

In 2018, the department of education of the administrative region of the Canton Vaud in Switzerland decided to integrate educational robots as one of the means to teach core CS concepts from primary school onward. The endeavour aims to initiate all teachers to the new discipline, irrespective of their initial perception and interest in either CS or robotics, and achieve so by means of an adequate professional development program. The intent of the study is therefore two-fold. From a practitioners’ perspective, we evaluate the introduction of educational robotics into formal education through CS curricular reform, from the lens of adoption and sustained adoption (i.e. the short and long term integration of the content into teacher practices), a facet seldom explored in the literature on ER professional development (Schina et al., 2020). This will help determine on the one hand whether CS curricular reform is a viable avenue for roboticists to introduce ER activities into formal education, and on the other hand the extent to which CS as a discipline benefits from the additional ER activities through the evaluation of teacher practices. From a researchers’ perspective, we also contribute to filling a gap in the study of Continuing Professional Development (CPD) programs, by assessing teachers’ adoption of the proposed ER activities and bridging it with their perception of the same activities. The underlying research questions are the following:

- **RQ1:** To what extent do teachers adopt, i.e. freely decide to introduce, the ER activities in their practices with respect to the overall CS activities proposed in the CPD program?
- **RQ2:** How does the introduction of ER through CS Education impact teachers’ perception of ER, specifically in light of their background and their prior experience with ER? How does this influence their adoption of the CS content?

Our study, involving a cohort of approximately 350 K-4 teachers, suggests that 1) ER not only benefits from CS curricular integration, but is also pivotal to the integration of CS as a discipline, and 2) primary school teachers, irrespective of their background and prior experience, perceive the benefits of ER and are open to integrating it into their practices.

## Related work

In Section 2.1 we recap the literature on teacher training efforts to introduce ER into teaching practices. Section 2.2 revises existing connections between ER and CS, while Section 2.3 explores the specific case of primary school and the role ER plays in current primary school CS curricula.

### Educational robotics professional development endeavours

Robots have been traditionally proposed in educational contexts at two levels: as tools manipulated by students during purposely-designed learning activities, or as social agents acting as peers for the students, or assistants for the teachers, during (possibly) traditional learning activities. The former are mostly known as Educational Robots, while the latter are more commonly referred to as Social Robots for Learning. Studies that focus on perception, which is highly linked to the second order internal barriers evoked by Ertmer (2005), are far less numerous in the case of ER than social robots for learning (Kennedy et al., 2016; Reich-Stiebert & Eyssel, 2016; Serholt et al., 2017). This is despite the fact that a positive attitude towards ER is a necessary stepping stone that must be achieved through teacher training if teachers are expected to introduce educational robotics into their classes (Negrini, 2020). Moreover, studies that assess ER perception are often within the context of voluntary workshops and professional development programs that suffer from a lack of generalisability. Often the participants had a vested interest in ER to begin, as with Castro et al. (2018) who trained voluntary teachers, or, as in the case of Negrini (2020) as the teachers had just been introduced to robotics. Nonetheless, these studies seem to indicate that teachers perceive the potential of ER.

Other limitations in the literature on ER professional development (Castro et al., 2018; Schina et al., 2020) include the fact that “training courses for teachers are not a common practice and reports of such sporadic activities are often inconclusive” (Castro et al., 2018). There are also few cases of ER professional development involving primary school teachers (Castro et al., 2018), although they would be the best suited to exploit the transversal nature of ER in their teaching and despite the benefits of an early introduction to ER (see Section 2.3). And finally, few studies address the question of the integration of the content (referred to as adoption) into teacher practices (Schina et al., 2020).

One example of a CPD involving primary school teachers is the study by Chevalier et al., (2016) where 44 teachers (including 24 primary school teachers) that had participated in at least one ER training session were surveyed. Their findings indicated that the teachers perceived robots as beneficial to promote reflection and collaboration, alongside other transversal skills such as communication, learning strategies and creative thinking. Similarly, Khanlari (2019) surveyed the 58 participants of an 8-h ER training session for primary school teachers. The teachers showed improved perception of robotics, “highlight[ing] the importance of learning about robotics and being engaged in hands-on activities with robotics” to improve their perception of robotics. Finally, Scaradozzi et al. (2019) introduced 184 in-service teachers to ER, coding and tinkering. They evaluated basic knowledge and self-efficacy and reported significant improvements in both domains. These findings confirm at a larger scale those of Jaipal-Jamani and Angeli (2017) who found that 21 in-service teachers significantly increased their content knowledge, interest and self-efficacy with robotics after a 6-h robotic intervention. Interestingly, while the above studies demonstrate their effectiveness in addressing second order barriers pertaining to perception, they do not provide any indication related to what the teachers actually did in their classrooms after the CPDs.

While a number of ER-CPDs validated teachers’ participation (Schina et al., 2020) by asking the teachers to deliver an ER session to students (Hynes & Santos, 2007; Sullivan & Moriarty, 2009; Conchinha & Freitas, 2015; Hodges et al., 2016; Leonard et al., 2017), only few studies assess the integration of ER into teachers’ practices following the program. Kay and Moss (2012) used a follow-up survey after a summer workshop with 20 teachers, to estimate that approximately 270 students had been exposed to the content as a consequence of the training program. Negrini (2019), who conducted, over two years, a robotics training program with 17 voluntary teachers from early childhood education and primary school, generally reported that “most of the participating teachers [had] integrated robotics into their annual program”. At a larger scale, Castro et al. (2018) conducted a 32-h module spread out over 8 months with 339 teachers from different grades that had already been active in ER for several years. Among the 254 who completed the post survey, 156 reported having conducted an ER experience during the professional development. In both cases, anyway, no information is given about what the teachers integrated in their practices, how and to what extent, nor about the factors that influenced their decision.

To summarise, the literature highlights the lack of ER teacher training programs, with only a few being conducted with teachers who were not already interested in robotics prior to the start of the program. However, if the objective is to introduce ER into formal education with teachers who are novices in ER and likely less interested in the topic, it is of paramount important to investigate whether the findings from studies with volunteer participants hold in the general case as well. Furthermore, the limited attention paid to assessing the impact of CPDs on teachers’ practices is detrimental to understanding teachers’ adoption of the proposed activities and factors influencing it. We believe that gaining clear insight into what makes a teacher decide to adopt ER activities into their practices is pivotal to improving ER-related professional development programs and the successful integration of robotics into formal education.

### Educational robotics to teach computer science

While robotics was once primarily used to teach about robotics, it has recently expanded towards other subjects (Jung & Won, 2018). In particular, as stated by Daniela and Lytras (2019), “educational robotics (ER) is [nowadays] mostly associated with Science, Technology, Engineering, and Mathematics (STEM)” (Alimisis, 2013; Castro et al., 2018; Eguchi, 2014; Greca Dufranc et al., 2020; Jaipal-Jamani & Angeli, 2017; Martín-Páez et al., 2019). Unfortunately, the association with STEM has not been a lucky choice for ER, given that “STEM education in schools appears to be inadequate” (Castro et al., 2018, citing Osborne & Dillon, 2008) and “an effective introduction of new technological tools in schools is lacking” (Castro et al., 2018, referencing Alimisis, 2013). Indeed, in their systematic review around ER in STEM, Benitti and Spolaôr (2017) found that “most of the selected papers fall into the extracurricular or hybrid category” with only 18% of them reporting formal applications of ER. This association between ER and STEM being neither indispensable, nor, it seems, particularly effective, pushes us to re-evaluate this decision by considering which disciplines would benefit most from employing ER as a means to teach the related content. Recent studies have employed ER as a medium to teach computational thinking (CT) skills (Angeli & Valanides, 2020; Atmatzidou & Demetriadis, 2016; Chalmers, 2018; Constantinou & Ioannou, 2018; Leonard et al., 2016). Chevalier et al., (2020) even developed a model of creative computational problem solving skills to assess the thought process of students engaged in ER activities. While the objective of the model is to help teachers design, implement and assess ER activities in order to foster relevant CT and creative problem solving skills, it also effectively demonstrates the benefits of ER for this domain. Similarly, an increasing number of CS-specific curricula and studies employ educational robots as a medium to teach CS concepts (Magnenat et al., 2014; Roy et al., 2015; Chevalier et al., 2016; Spolaôr & Benitti, 2017; Elkin et al., 2018; Bers, 2019), often through programming. This is partly because this “embodied IDE” is more engaging than alternative virtual interfaces (Mubin et al., 2013). CS, however, should not be reduced to programming. The decomposition into 1) Algorithmics and programming, 2) Information and data, 3) Machines, systems and networks put forth by Schiper (2016) helps consider robotics within CS as a machine composed of actuators and sensors, which relies on algorithms and programs to connect its perception to its actions.

Therefore, despite the frequent association of ER with STEM, with many countries looking to or having already integrated CS into their curricula (Balanskat & Engelhardt, 2015; Bocconi et al., 2016; The Committee on European Computing Education (CECE), (2017); European Union & Education, 2019; Falkner et al., 2019), we believe that CS Education is a promising avenue for the integration of ER into formal education. Not only does a combined CS and robotics curricular reform remain in line with the objectives of the CS Education curriculum, but it may even facilitate the introduction of more STEM related activities into formal education in the long run.

### Educational robotics in formal primary school education

There are numerous advantages to introducing robotics in early childhood years, ranging from improved engagement, developing fine motor skills, hand eye coordination, engaging in collaboration and teamwork (Bers et al., 2002, 2014). Students learn about “powerful ideas of engineering, technology, and computer programming while also building […] computational thinking skills” (Bers et al., 2014) and helping combat gender stereotypes in technical domains (Sullivan & Bers, 2019). Indeed the “research suggests that children who are exposed to STEM (Science, Technology, Engineering, and Mathematics) curriculum and programming at an early age demonstrate fewer gender-based stereotypes regarding STEM careers (Metz, 2007; Steele, 1997) and fewer obstacles entering [related] fields (Madill et al., 2007; Markert, 1996)” (Sullivan & Bers, 2018). Despite these benefits, few countries include ER as a means to enhance learning of CS or STEM related concepts in their curriculum, and even less do so at the level of primary school. As an example, only 5 among the 21 European countries considered by the survey of Balanskat and Engelhardt (2015) integrate robotics within their CS curricula, and only Slovakia reported using ER at the primary school level to learn how to program and control robots. Additionally, the way robotics is integrated into the curriculum varies greatly, not just due to the way the scope pertaining to ER is defined, but also due to the way the curriculum is reinforced in the different countries, sometimes giving more or less freedom and flexibility to individual institutions and teachers in their interpretation of the curriculum. In general, the role of ER in primary school is even less prominent than in upper secondary school, in big part because CS and Digital Education is globally less present at that level (European Union & Education, 2019).

Provided the benefits of an early introduction to ER, and despite the numerous ongoing Digital Education reforms, few countries seem to be capitalising on this knowledge and introducing robotics as a means to teach core CS concepts in primary school. Conjointly with the high variability in the CS curricula being introduced internationally and the varying degrees of liberty of teachers, it is essential to gain insight into the role ER can play in CS Education and the interplay between them. The analysis must place teachers at the centre as they “are the linchpin in any effort to implement or change [in computing education]” (Blikstein & Moghadam, 2019) and consider both their perception and what they actually implement in classrooms. Capitalising on such knowledge will help guide future endeavours and provide teachers with both adequate pedagogical content knowledge and resources to introduce both ER and CS in formal education. Hence, we deem the analysis of the interplay between CS and ER to be timely and relevant for shaping the future of ER in formal education, notably when considering an integration at the level of primary school.

### Present study

As the literature review above outlines, the integration of Educational Robots into formal education at the level of primary school is still an open challenge, despite the numerous proofs of its significance and benefits. Similarly, few studies have been able to analyse the short- and long-term adoption of ER activities by in-service teachers, as a follow-up of teacher training and professional development programs, even though adoption is a crucial metric for establishing the true success of teacher training programs. With this study, we contribute to addressing both issues.

## Methods

### Context

The CS curricular reform for the first four grades of primary school (students aged 4–8 years old) considered in the present study, and the corresponding CPD program, are part of a larger Digital Education reform put forth in the Canton Vaud in Switzerland (El-Hamamsy et al., 2020). The CS curriculum was conceived around the three axes defined by Schiper (2016) (Algorithmics and programming; Information and data; Machines, systems and networks) and includes Educational Robotics as one of its means of teaching core CS concepts (El-Hamamsy et al., 2020). The CPD program was organised as a two-year endeavour, with the first year devoted to Computer Science (CS) and the second year to Information and Communication Technologies (ICT) alongside Digital Citizenship. The evaluation of teachers’ CS practices was carried out in both years. Accompanying personnel was concurrently trained to support the teachers in their implementation of the content in the classrooms.

The in-service CS-CPD program, which is interestingly aligned with the recommendations for ER teacher training recently proposed by Schina et al. (2020), was based on a set of training principles (El-Hamamsy et al., 2020) first elaborated by Chessel-Lazzarotto (2018): it is a collaborative, hands-on CPD that encouraged teachers to practice the content by providing them resources and materials for implementing the content in their classrooms, alongside long-term support in their institutions (El-Hamamsy et al., 2020). While no condition was explicitly set to validate teachers’ participation in the mandated in-service program, voluntary adoption of the content seen in the CPD, both in the short and long term, was employed as a metric to validate the success of the program.

### Structure of the CS-CPD

The CS-CPD was designed to introduce teachers to core CS concepts in four training sessions spread over the whole school year, so that teachers would have time to introduce the content into their practices and reflect on it. Throughout the sessions, the teachers were progressively introduced to “1) CS Unplugged activities, to discover the basics of algorithmics, 2) Robotics Unplugged activities^1^ [(i.e., that involve the use of the physical robot without screens)], to learn about the different components of machines (sensors, actuators) and their behaviours, 3) more advanced CS concepts and visual programming activities and finally 4) advanced concepts in algorithmics, information and data structures, together with elements of creative computing (CS and arts)” (El-Hamamsy et al., 2020). A total of 13 student activities that are directly transposable to the classroom were proposed during the sessions (see Table 1): 9 Computer Science Unplugged (CSU) activities, 2 Robotics Unplugged (RU) activities, 1 Robotics Visual Programming (RVP) activity and 1 (non-robotic) Visual Programming (VP) activity. The CS concepts addressed by the proposed activities are summarised in Table 2. As the table shows, the Educational Robotics learning activities were designed to include a wider and richer range of concepts and pedagogical sequences than the CSU counterparts, which explains and compensates for their lower proportion. Moreover, the table shows that the ER activities can cover a big part of the CS concepts in the curriculum, reinforcing the role that ER can play within CS education.

**Table 1:** Summary of the 13 student activities proposed during the CS-CPD program

Activity	Activity type	Recommended duration of an in-class session in periods (45-min units)	CS-CPD session
The sorting machine	CSU	4	1
The robot game	CSU	4	
The crane game	CSU	4	
The pixel game	CSU	4	
Treasure hunt	CSU	5	
Bluebot	RU	6	2
Pre Programmed Thymio	RU	4	
Thymio VPL	RVP	2	
Daily algorithms	CSU	4	3
Salmon sorting	CSU	1	
Networks	CSU	2	
Scratch Jr	VP	5	
Cryptography	CSU	1	4

**Table 2:** Concepts covered by the proposed activities in relation to the domains defined by Schiper (2016). Each domain is decomposed into concepts. Algorithms and Programming covers Algorithms (A1), Language (A2), Instructions (A3), Programs (A4), Conditions (A5), Loops (A6) and Debugging (A7). Machines and Networks covers the notion that machines execute orders (M1), the components of machines and robots (M2), the notion that robots have sensing-to-action loops (M3) and that machines can be connected into networks (M4) that need security measures (M5). Information and Data covers the notion of Encoding (I1)

Activity	Activity type	Algorithms and Programming							Machines and Networks					Information and Data
		A1	A2	A3	A4	A5	A6	A7	M1	M2	M3	M4	M5	I1
The sorting machine	CSU	x		x		x	x		x					
The robot game	CSU	x	x	x	x		x		x					
The crane game	CSU	x	x	x	x	x	x	x						
The pixel game	CSU		x											x
Treasure hunt	CSU			x	x	x								
Bluebot	RU	x	x	x	x	x	x	x	x					
Pre Programmed Thymio	RU				x	x		x		x	x			
Thymio VPL	RVP	x	x	x	x			x	x	x	x			
Daily algorithms	CSU	x	x	x	x	x	x							
Salmon sorting	CSU	x		x		x	x		x			x		
Networks	CSU											x	x	
Scratch Jr	VP	x	x	x	x	x	x	x	x					
Cryptography	CSU												x	

### Participants and data collection

Nearly 350 teachers of grades 1–4 (i.e., all of the teachers employed in 10 schools in the lead by the minister of education of the Canton, selected as pilot institutions for the CPD program) participated in the CPD program and the evaluations reported in this study. At the end of each training session the teachers were administered a survey to assess their perception of the CPD program and their adoption of the content. Details about the response rates we obtained are provided in Table 3.

**Table 3:** Number of teachers participating in the data collection, by training session and grade. Grades 1 and 2 are denoted by 1-2P, and grades 3 and 4 by 3-4P. The category “Other” includes teachers that are not from the cycle, specialised teachers, and teachers for whom the information is missing (a common case especially in session 1). The total number of responses is provided, as well as (for Year 2) the total number of consistent responses, i.e. with matching IDs, which are used for the longitudinal adoption analysis

Year	Training Session	1-2P	3-4P	Other	Total	Total Consistent
Year 1	CS—Day 1 (Oct. 2018)	85	96	110	291	-
	CS—Day 2 (Nov. 2018)	130	136	54	320	-
	CS—Day 3 (Mar. 2019)	124	137	45	306	-
	CS—Day 4 (Apr. 2019)	66	111	37	214	-
Year 2	ICT (no CS)—Day 6 (Dec. 2019)	146	159	19	324	181
	ICT (no CS)—Day 7 (Mar. 2020)	128	150	20	298	181
Year 2	End of program survey—(Jun. 2020)	37	41	6	69	-

#### Surveys during the CS professional development program

During the CS-CPD training (first year of the CPD program), the survey administered to teachers at the end of each training session addressed their perception of the session (Perception items in Table 4) and the time spent integrating in their classroom content seen in previous sessions (Adoption items in Table 4). As previously stated, while the teachers were encouraged to integrate the proposed activities in their classrooms, they were not forced to do so. For this reason, surveying the teachers’ voluntary adoption of the content within the mandatory professional development program is a revealing metric of its success.

**Table 4:** CS-CPD survey items. Adoption data of session 4 is discarded due to a data collection error

Topic	Question	Session
Contextual factors	Grade taught	1–4 & 6–7
Perception (4-point Likert scale)	The training sessions were rich and interesting	1–4
	The level of difficulty was well adapted	
	The equilibrium between theory and practice was well adapted	
	I appreciated the content seen in the training session	
Adoption	How many periods did you do per activity? (13 activities)	2–3 & 6–7

#### End of program survey

A final training session set for May 2020 was cancelled due to COVID-19. In its place, an end-of-program survey was electronically administered, to assess the whole CPD program. This survey included an assessment of the adoption of the proposed CS activities over the course of the second training year, as well as an in-depth assessment of teachers’ perception of robotics (see Table 5). Drawing inspiration from intrinsic motivation theory (Ryan & Deci, 2000), the concept of interest (and inversely reticence) with respect to adopting ER is considered. As acceptance of technology innovation theories (and in particular Technology Acceptance Models, Davis, 1989) include ease of use and perceived usefulness as predictors for behavioural intention and subsequently usage (King & He, 2006), both self-efficacy and utility are employed in the analysis. Utility is considered with respect to CS, other disciplines, transversal skills and student attitudes, similarly to what was done by Castro et al. (2018). Finally, as recommendation has been found to be highly correlated with a customer’s return in the literature on customer satisfaction (Danaher & Haddrell, 1996), this is used as an indicator which should correlate highly with adoption. Since this survey was conducted outside of the training sessions, and elicited much lower response rates, it is considered separately in the analyses.

**Table 5:** End of program survey items

Topic	Question	Answer format
Contextual factors	Grade taught	Checkboxes
	Age, teaching experience, prior ICT experience, prior robotics experience	Years
	Gender	Checkboxes
Adoption	Over the course of this second year, what activities did you adopt?	Checkboxes
Interest in ER	I want to integrate robotics activities in my classroom I’m interested in integrating robotics into my long term practice	Likert
Positive ER self-efficacy	I feel able to conduct robotic activities in my classroom I feel capable of creating a robotics instructional sequence	Likert
Negative ER self-efficacy	I think integrating robotics into my practice is difficult I think it’s time-consuming to integrate robotics into my practice I need support in the classes where I use educational robots	Likert
ER adoption reticence	I’m reluctant to integrate robotics into my classes	Likert
ER recommendation	I would recommend robotics as an educational tool to other teachers from my grade	Likert
ER utility for CS	I think robotics is useful for teaching computer science	Likert
ER benefits for other disciplines	I believe robotics is useful for the following disciplines: *Maths, science, French, history, sports, art, none*	Likert
ER benefits for transversal skills	I believe robotics is useful to help students develop the following transversal skills: *Creativity and imagination, critical thinking, problem solving, verbalization, argumentation, inter-personal communication, collaboration*	Likert
ER benefits for student attitudes	I believe robotics can positively influence the students in term of the following attitudes: *Motivation and engagement, interest, curiosity, confidence, fear of error, reflection, focus, perseverance / endurance, anticipation, autonomy, responsibility*	Likert

Likert denotes items evaluated on a 4-point Likert scale, with 1 = Totally Disagree and 4 = Totally Agree

### Adoption analysis

The analysis of teachers’ adoption is conducted from the perspectives of:

1. The overall number of periods (teaching sessions of 45 min) conducted by the teachers per type of activity, and its evolution over the two years of the CPD program.
2. The proportion of adopters per type of activity, and its evolution over the two years of the CPD program.
3. The adoption seriousness per type of activity, and its evolution over the two years of the CPD program. The term seriousness is inspired from Dewey, who highlighted the importance that teachers engage in both playful and serious learning experiences. While “playful [refers to engaging] the interest of the student, [and opening] up students to the possibility of new knowledge […] serious [interactions help ensure that] learning is absorbed in terms of clearly identifiable ends” (Skilbeck, 2017). The adoption seriousness metric is based on a relative grading approach which ranks teachers based on a proxy for quantity (the number of different activities conducted), completion (the number of activities conducted for a sufficient duration to have a meaningful pedagogical sequence) and the frequency with which the activities were implemented in the classrooms. These metrics relate to traditional e-learning assessment rubrics pertaining to the completion of a task, score and timing. Concretely, teachers that have not adopted any of the proposed activities are attributed rank 0. The remaining teachers are relatively ranked in terms of adoption seriousness from 1 to 3 with 1 being the bottom third, and 3 being the top third. The analysis is conducted for both the first and second year and provides additional insights as to the extent to which teachers adopt a given type of activity.

## Results

To evaluate the interplay between ER and CS within a CS-CPD that employed robotics as one of the means to teach CS (see Section 3), we consider 1) whether CS curricular reform is a viable avenue for educational roboticists to introduce ER activities in formal education, and 2) the extent to which CS as a discipline benefits from the additional ER activities through the evaluation of teacher practices. As stated previously, the research questions guiding the analysis are:

- **RQ1:** To what extent do teachers adopt, i.e. freely decide to introduce, the ER activities in their practices with respect to the overall CS activities proposed in the CPD program? This analysis provides not only an indication as to how CS should be operationalised but also about which instruction modalities are most adapted to incarnate the underlying notions. Moreover, short- and long-term adoption analysis is a missing metric for most CS and ER CPD programs and training initiatives, which we deem crucial for improving their design and ensuring their effectiveness.
- **RQ2:** How does the introduction of ER through CS Education impact teachers’ perception of ER, specifically in light of their background and their prior experience with ER? How does this influence their adoption of the CS content? This analysis, bridging between teachers’ perception of ER and adoption of ER activities, concretely aims at investigating the relationship between a solid, but rarely accessible metric (adoption) and a well-established evaluation mean for CPD programs (perception).

### RQ1: A comparative analysis of the adoption of non-ER versus ER activities

As detailed in Table 2, the CS-CPD included 3 ER activities (two RU activities and one RVP activity) and 10 non-ER activities (nine CSU activities and one VP activity). The two Visual Programming activities, while accounting for more than 15% of the proposed activities, represent less than 10% of the teachers’ adoption time in both years of the program. The low adoption of these activities seems to echo the reticence mentioned by teachers in Negrini (2020)’s study regarding young children spending too much time in front of screens. That is why CSU and RU activities, that do not require screens to learn the underlying concepts, are all the more relevant in the present context which targets the first four grades of primary school. As a consequence, we focus the following analysis on RU and CSU activities. Beside a longitudinal comparative adoption analysis over the two-year CPD program, we also consider specific facets of adoption, namely: number of periods, proportion of adopters per activity type, and adoption seriousness.

#### Longitudinal adoption analysis—number of periods per activity type

All RU activities were seen in session 2 of the CS-CPD program, which was perceived as the most interesting by the teachers (Kruskal Wallis test p < 0.001, H = 14 compared to session 1, H = 44 compared to session 3 and H = 28 compared to session 4, El-Hamamsy et al., 2020). However, the results described below suggest that teachers, while immediately interested by RU activities, required more time compared to CSU to appropriate the content and conduct the activities in their classrooms.

**Fig. 1:**
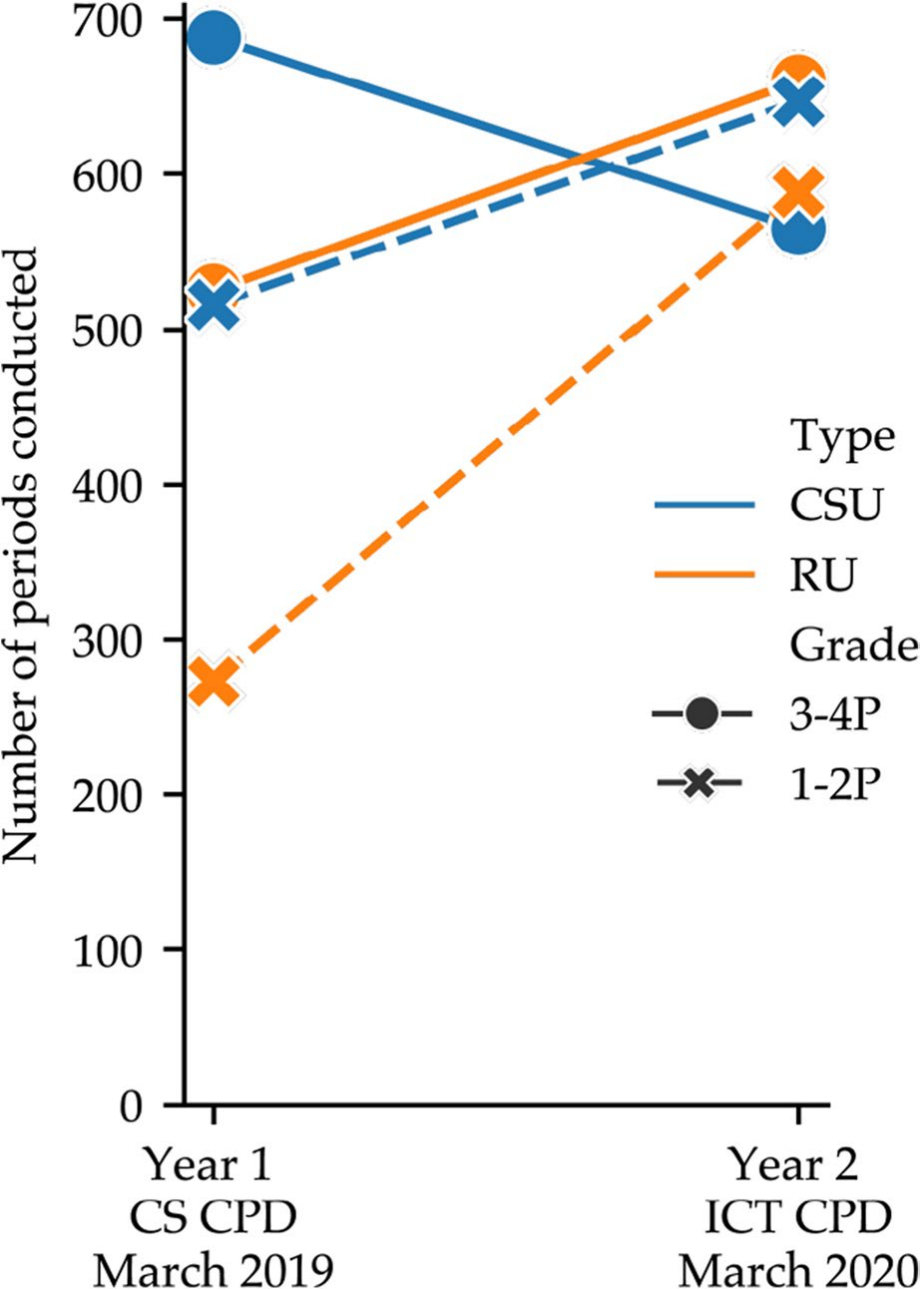
Number of periods conducted by the teachers per activity type from the beginning of each school year

Figure 1 shows the evolution of the overall number of periods conducted per grade (1-2P versus 3-4P, line type and marker) and activity type (CSU versus RU, line colour). In year 1, the overall number of RU periods (orange) is lower than that of the CSU activities (blue) both for teachers in grades 1-2P (dashed line, students aged 4–6) and those in grades 3-4P (solid line, students aged 6–8). Moreover, teachers in 1-2P adopt nearly half as much as those in 3-4P. In year 2, there is a notable increase in the overall number of RU periods, for both grades and especially for 1-2P. While in Year 2 the leap in RU periods conducted by teachers in 1-2P was accompanied by an increase in the number of CSU periods (with CSU remaining the leading type of activity), teachers in 3-4P increased the number of RU periods conducted with respect to Year 1 and decreased the number of CSU periods. Indeed, RU is the leading type of activity for 3-4P teachers during Year 2.

As reported in Table 1, the designers of the CS-CPD envisioned pedagogical sequences that allow students to appropriate the underlying CS concepts which for CSU entailed conducting pedagogical sequences of 3.2 periods on average, while RU activities were accorded 5.0 periods. The difference arises from the way ER activities are conceived, aiming to address a wider range of concepts through a single task. To verify whether and to what extent teachers agreed with the proposed sequences, the distribution of sequence length per activity type was computed during Year 2. Indeed, CSU activities were conducted in sequences of 2.9 periods on average, while RU activities took 4.6 periods on average, suggesting that teachers agreed with the provided pedagogical sequences and, most importantly, implemented the activities in their classrooms long enough for their students to appropriate the related CS concepts.

#### Longitudinal adoption analysis—number of adopters per activity type

**Fig. 2:**
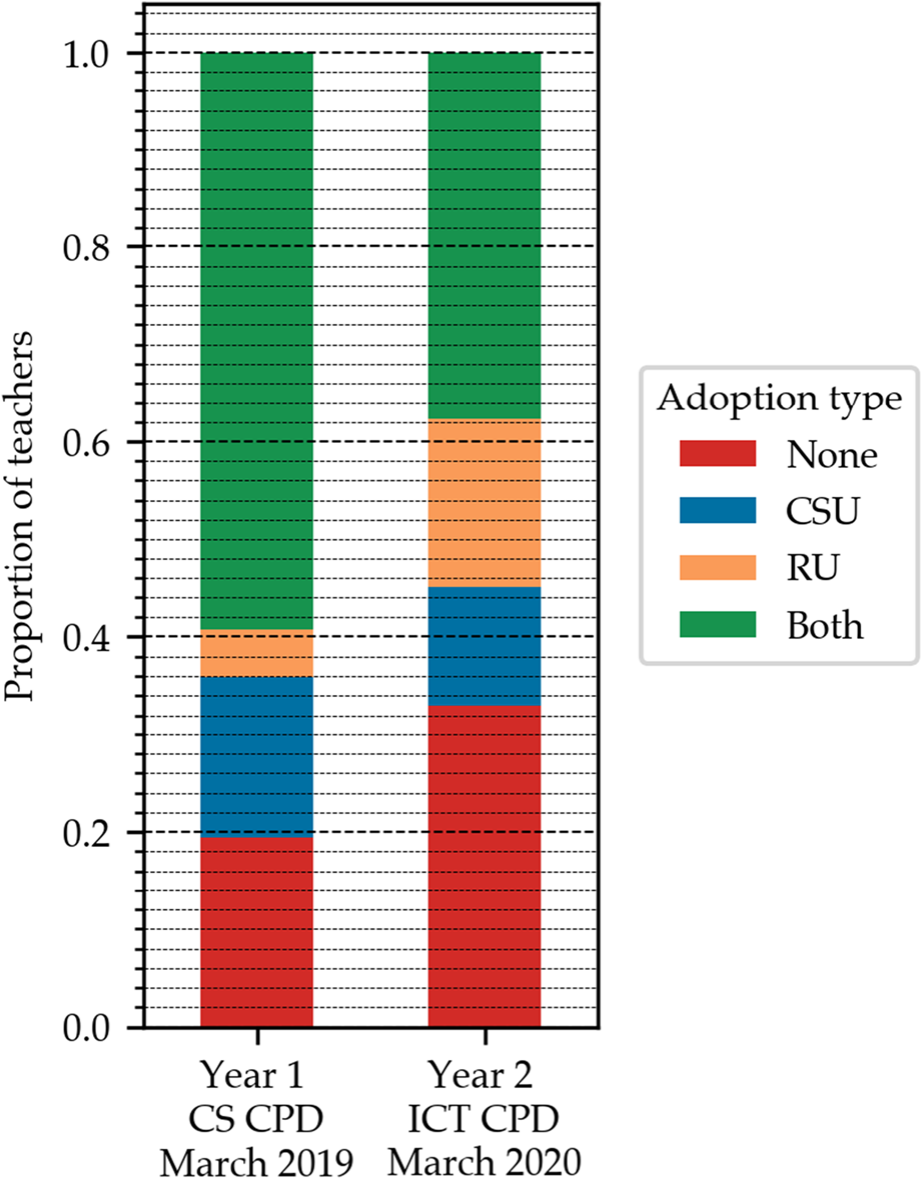
Evolution of adoption type over the course of the CPD program. To allow for comparison, adoption is sampled in March for both years, since the schools were closed between March and May 2020 due to COVID-19. Cochran’s Q test of independence for matched pairs of subjects on the observed counts for the adoption type per year is not significant

The analysis of the number of periods conducted neglects the number of teachers implementing any of the proposed activities in classrooms (i.e., the number of adopters). Figure 2 shows the evolution, over the two years of the CPD program, of the proportion of adopters, denoting teachers who did not adopt any of the proposed activities in red, those who only adopted CSU content in blue, those who only adopted RU content in orange and, finally, teachers who adopted at least one activity of both types in green.

In Year 1 over 80% of teachers conducted at least one CS activity, compared to 65% in Year 2. While a direct comparison between the first and second year rates cannot be made (notably since the decrease in adoption rates is likely related to the early interruption of the school year caused by the COVID-19 pandemic), relative changes can be assessed. As the Fig. 2 shows, in Year 1 there are more CSU adopters (76% of the total, sum of the green and blue area) than RU adopters (64%, sum of the green and orange area). Conversely, in Year 2 55% of the teachers adopts RU activities, while only 50% adopts CSU activities. The change is due to a noticeable, although not significant, shift towards only adopting RU activities (orange surface increasing from 5% in Year 1 to 17% in Year 2), matched by a decrease in the proportion of teachers only adopting CSU activities (blue surface decreasing from 17 to 12%). It would therefore be very interesting to see how the trend continues to evolve in Year 3, after the end of the CPD program.

#### Longitudinal adoption analysis—adoption seriousness

While adopting just one type of activity could be indicative of a lack of implication in the program, it could also be indicative of certain teachers manifesting a preference, all the while covering all the core CS concepts of the program. Similarly, teachers that adopt both types of activities may just be dabbling lightly in the curriculum, without actually conducting meaningful pedagogical sequences. To discriminate between these cases, we propose to analyse the adoption seriousness, which considers proxies for quantity, completion and frequency (see Section 3.4).

**Fig. 3:**
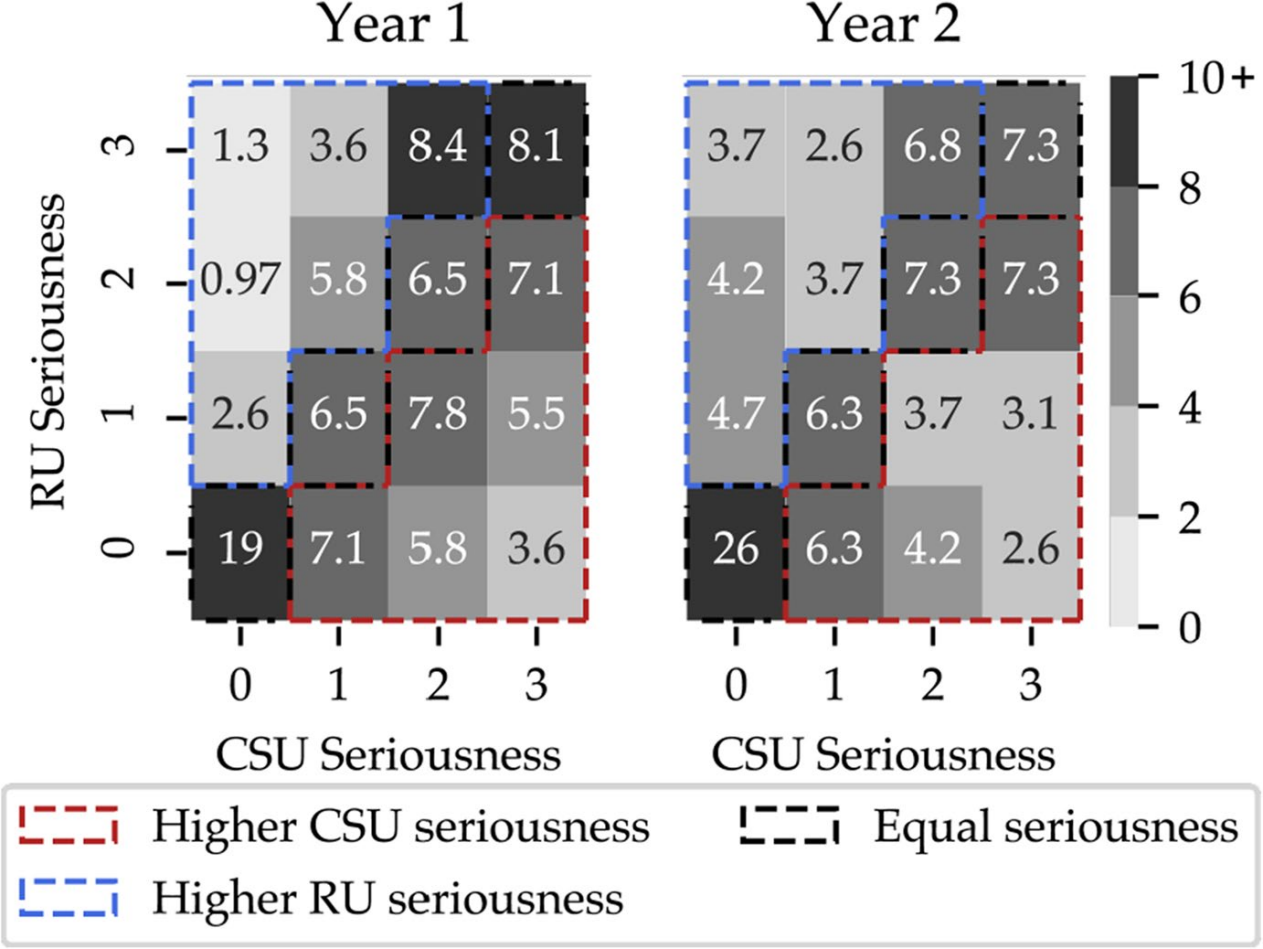
Distribution of the adoption seriousness of CSU versus RU activities in Year 1 (left) and Year 2 (right). In both matrices, a cell represents the proportion of teachers displaying the corresponding values of CSU and RU seriousness. As an example, in Year 1, 1.3% of the teachers included in the analysis displayed high seriousness in RU activities while not performing any CSU activity (s_RU_ = 3 and s_CSU_ = 0, top-left cell). This proportion increases to 3.7% in Year 2

Figure 3 shows the distribution of adoption seriousness for RU and CSU activities in the two years of the CPD program. Teachers tended to adopt the two types of activities with similar seriousness in Year 1 and 2 (delta seriousness = 1 for 79% of teachers in both years, Kolmogorov-Smirnoff test on the distribution of delta seriousness, p > 0.05), reinforcing the position of both types of activities in the curriculum. The teachers’ global seriousness (computed considering activities of all types) highly correlates with the RU and the CSU seriousness in both years (Spearman correlations ρ ≥ 0.75). In line with the other analyses, during Year 1 the global seriousness more strongly relates with the CSU seriousness (Spearman correlation ρ_CSU_ = 0.91; ρ_RU_ = 0.75), while during Year 2 RU seriousness and CSU seriousness seem to similarly contribute to the global seriousness (Spearman correlation ρ_CSU_ = 0.85, ρ_RU_ = 0.84).

It is interesting to note that the relationship between the two types of seriousness is moderate, suggesting that two types of activities are inherently different and possibly also appealing to different people. Indeed, 21.4% and 25.7% of teachers in Year 1 and Year 2, respectively, only conduct one type of activity, and some do so with very high seriousness. Specifically, in Year 1, 9.4% of the teachers only conduct CSU activities, and do so seriously (s_CSU_ = 2 or s_CSU_ = 3, with s_RU_ = 0), and 2.3% of the teachers are serious in conducting only RU activities (s_RU_ = 2 or s_RU_ = 3, with s_CSU_ = 0). In Year 2, this selective seriousness concerns 6.8% of teachers for CSU and 7.9% for RU activities. These results suggest the importance of diversifying the instruction modalities proposed to cover a same concept (see Table 2).

### RQ2: Analysis of teachers’ perception of robotics and its relation with adoption

Despite the low response rate of the end-of-program survey (69 complete responses), the results are reported for two reasons: 1) to ascertain whether the positive perception of robotics reported by other studies holds for non-voluntary primary school teachers following the CPD; and 2) to identify possible factors influencing the perception and adoption of robotics content. Lastly, 24% of the respondents of the end-of-program survey declared to have adopted none of the proposed activities (i.e., to be non-adopters). This percentage is close to the one extracted from the much bigger pool of respondents of the survey administered in the last session of Year 2 (33%, see Fig. 2), comforting us in the hypothesis that the respondents to the end-of-program survey are not only the highly motivated teachers, but to some extent representative of the larger group of teachers involved in the study.

#### Perception of ER

**Fig. 4:**
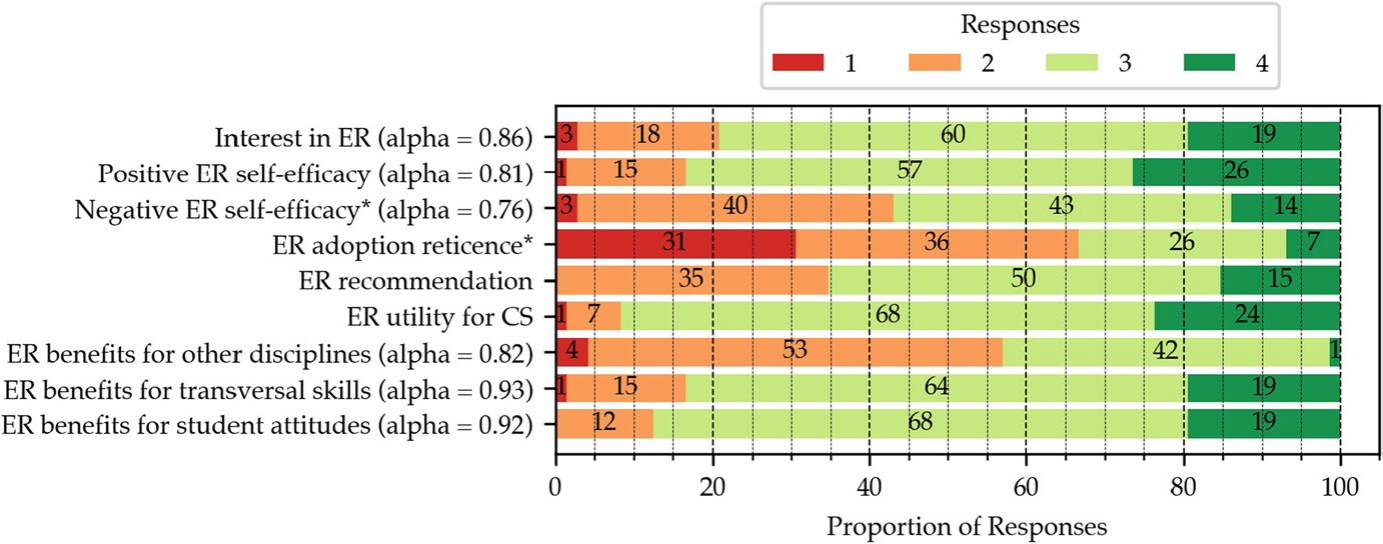
Distribution of the responses on the 4-point Likert scale (1—Totally Disagree, 4—Totally Agree) for the items pertaining to perception of ER. Constructs denoted by a * are negative items, therefore a 4 indicates that a teacher totally agrees with the negative statement. The questions are grouped into compound items based on the categorisation in Table 5 for conciseness. Cronbach’s α for internal consistency is provided for the constructs composed of multiple questions

The results of the questions listed in Table 5 are presented in Fig. 4. The teachers want to conduct ER activities their classrooms (compound item Interest in ER) both in the short- (78% of “agree” and “totally agree” responses) and the long-term (62%). They believe they are capable of doing so (83%, compound item Positive ER self-efficacy, Cronbach’s α = 0.81), although a number of teachers reported that integrating ER into their practices is time consuming (76%), difficult (55%), and requiring support in the classroom (49%) (compound item Negative ER self-efficacy, α = 0.76). Despite these preoccupations, the perception of robotics is globally positive at the end of the two-year CPD: only 33% of teachers are reticent towards adopting ER and just 35% would not recommend ER to their peers.

In addition to being interested and confident in their capacity to introduce robotics into their practices, the teachers perceived ER as useful to teach CS (93%) and even other disciplines (74%, compound item ER benefits for other disciplines). This is notably the case for maths (98%), science (75%) and more surprisingly French (61%), and arts (41%). Furthermore, there seems to be a consensus around the utility of ER in terms of transversal skills (83%, compound item ER benefits for transversal skills), notably collaboration (89%) and problem solving (91%). Similar results were obtained concerning student attitudes (88% of positive responses) with interest, curiosity, motivation and engagement being perceived as positive outlets by over 90% of teachers.

#### Relation between perception, recommendation and adoption of ER activities

The connection between perception and adoption, while undoubtedly existing, is not straightforward. A teacher who has a positive perception of ER might be prevented from adopting by one or more of the other first and second order barriers (Ertmer, 2005), while another one might be motivated to adopt by external factors which have nothing to do with perception (through a form of controlled, or extrinsic motivation,^2^ Ryan & Deci, 2000; Ryan and Deci, 2020), and even despite a negative perception of ER. In this work, we consider recommendation as a metric related to both perception and adoption (Danaher & Haddrell, 1996), and thus helpful to untangle the relationship between the two constructs.

When asked whether they would recommend robotics as an education tool to other colleagues, 35% of the respondents said that they would not recommend it, while 50% would and 15% would even highly recommend ER to their peers. Table 6 analyses the coherence between teachers’ recommendation and, respectively, their adoption (first group of rows), prior experience and contextual factors (second group) and perception-related constructs (last two groups). When considering the number of activities implemented, and RU activities in particular, there are significant differences (p < 0.05 with large effect size) between those who would not recommend and those who recommend highly. The Not Recommend—Recommend comparison is in line with this finding, albeit not significantly. The extent to which a teacher recommended robotics thus appears to be positively correlated with their adoption of ER, and as such overall adoption. At the same time, the Table shows that recommendation is also strongly linked with perception, with significant differences being reported between all recommendation groups on interest, self-efficacy, utility and perception of benefit.

**Table 6:** Comparison between recommendation responses and adoption, prior and contextual factors, perception constructs (Kruskal–Wallis test). Comparisons are conducted between those who would not recommend, those who would recommend and those who would highly recommend

Recommendation of ER to other teachers	Not Recommend vs. Recommend	Not Recommend vs. Highly Recommend	Recommend vs. Highly Recommend
Number of activities	-	*, H = 5.8, D = -0.95	-
Number of RU activities	-	*, H = 5.1, D = -0.86	-
Number of CSU activities	-	-	-
Gender	-	-	-
Age	-	-	-
Grades taught (1-2P versus 3-4P)	-	-	-
Years of teaching experience	-	-	-
Years of ICT experience	-	-	-
Years of robotics experience	-	-	-
Interest in ER	***, H = 15.6, D = -1.14	***, H = 17.4, D = -2.18	**, H = 6.8, D = -0.97
Positive ER self-efficacy	**, H = 7.7, D = -0.79	***, H = 13.5, D = -1.69	*, H = 4.5, D = -0.79
Negative ER self-efficacy	***, H = 12.7, D = 1.05	***, H = 19.0, D = 2.08	*, H = 4.1, D = 0.8
ER adoption reticence	***, H = 13.1, D = 1.01	***, H = 15.9, D = 2.04	-
ER utility for CS	**, H = 6.6, D = -0.71	***, H = 14.6, D = -1.37	*, H = 5.8, D = -0.73
ER benefits for other disciplines	-	***, H = 15.7, D = -1.88	**, H = 9.7, D = -1.28
ER benefits for transversal skills	**, H = 9.7, D = -0.68	***, H = 15.8, D = -1.94	**, H = 10.7, D = -1.27
ER benefits for student attitudes	**, H = 9.4, D = -0.8	***, H = 16.0, D = -1.89	**, H = 7.9, D = -1.13

Non-significant p-values are denoted by “-”, whilst significant values with p < 0.05 are denoted by *, p < 0.01 by ** and p < 0.001 by ***. Significant values are accompanied by the corresponding Kruskal Wallis H statistic and Cohen’s D for effect size. An effect size around 0.2 is considered small, around 0.5 medium and 0.8 large

#### The influence of prior experience with ER

Interestingly, ER recommendation seems to be completely unrelated to age, gender, grades taught, prior teaching experience, prior ICT experience and even prior experience with Educational Robotics. To further verify this finding, we ran the complementary analysis, by separating teachers with prior experience with ER (the pioneers, 17 respondents) from those without (the novices, 55 respondents) and checking for differences between these two groups on the afore-discussed items of adoption, prior and contextual factors, and perception. The results of this analysis are reported in Table 7. Prior experience with ER appears to be unrelated to experience with ICT, teaching, age, gender or the grades taught, suggesting that any teacher could be an ER pioneer. Moreover, there seems to be no significant difference between pioneers and novices concerning their interest, perceived utility (with the exception of interdisciplinary links) and even the adoption of the ER activities. These findings suggest that the CPD program was successful in getting novices interested in ER and willing to integrate it in their practice. At the same time, pioneers have significantly higher self-efficacy than novices, likely due to having already conducted ER activities in their classrooms in the past, and better perceive the utility of ER for disciplines other than CS.

**Table 7:** Comparison between prior ER experience responses and adoption, prior and contextual factors, perception constructs (Kruskal–Wallis test). Comparisons are conducted between pioneers (those with prior ER experience) and novices (those without)

Prior Experience with ER	Novices vs. Pioneers
Number of activities	-
Number of RU activities	-
Number of CSU activities	-
Gender	-
Age	-
Years of teaching experience	-
Years of ICT experience	-
Interest in ER	-
Positive ER self-efficacy	*, H = 4.3, D = -0.61
Negative ER self-efficacy	**, H = 7.5, D = 0.8
ER adoption reticence	*, H = 5.3, D = 0.69
ER utility for CS	-
ER benefits for other disciplines	**, H = 10.2, D = -0.92
ER benefits for transversal skills	-
ER benefits for student attitudes	-
ER recommendation	-

Non-significant p-values are denoted by “-”, whilst significant values with p < 0.05 are denoted by *, p < 0.01 by ** and p < 0.001 by ***. Significant values are accompanied by the corresponding Kruskal Wallis H statistic and Cohen’s D for effect size. An effect size around 0.2 is considered small, around 0.5 medium and 0.8 large

Finally, in Table 8 we analyse whether pioneers and novices differ in the type of activities they adopt (only CSU activities, only RU activities, neither or both). Fischer’s exact test of independence fails to reject H0 (p > 0.05) therefore indicating that the adoption type is independent from the teachers’ prior experience with Educational Robotics.

**Table 8:** Adoption type with respect to prior experience with ER

	Neither	CSU	RU	Both	Total
Novices	15	4	4	32	55
Pioneers	2	3	2	10	17
Total	17	7	6	42	72

## Discussion

### On the adoption of ER activities in a CS-CPD

#### A delayed and growing adoption of robotics unplugged activities

Figure 1 shows that the amount of time teachers devoted to implementing unplugged activities in their classrooms increased over the two-year program. Moreover, the adoption of RU activities surpassed that of CSU activities in the second year of the program, despite an imbalance in the program towards CSU activities (see Table 1). These results suggest that teachers require more time to appropriate the RU content than the CSU counterparts and this finding, if confirmed by focused studies, would be of great importance for the design and assessment of CPD programs centred on or including Educational Robotics. This seems to echo the findings of Chevalier et al., (2016) who found that teachers who had not made use of educational robots before were more likely to believe that they as a teacher needed computer science skills to use the robot, therefore delaying the robot’s adoption compared to CSU activities in our case. Moreover, the presence of age and gender stereotypes surrounding the discipline is a well-documented fact (Clayton et al., 2009; King et al., 2002) which lead to “computer anxiety”. It is thus not surprising to find that the primary school teachers, who are mainly middle-aged women in our case, take more time to introduce robotics-based activities into their practices, compared to the CS Unplugged-type content which are much closer to their practices (El-Hamamsy et al., 2020). At the same time, the increasing time spent conducting RU activities could be due to their design, which featured extensive range of scenarios that the teachers could adapt and build upon. The longer and more articulated ER activities might have interested teachers more than the inherently shorter and narrower CSU sequences which tend to target a specific concept. If confirmed by future, focused analyses, this finding could be of great importance for the design of ER and CS activities to better align with teachers’ practices and increase the likeliness of adoption in their classrooms.

The positive trends observed over two years in terms of adoption, and especially adoption of ER activities, in a context where teachers were not forced to adopt, also indicate that the results were not driven by novelty. A continuous assessment of teachers’ adoption in the years after participation in the CS-CPD program will be fundamental to analyse the long-term impact and success of the program and the proposed activities, notably as teachers progress through stages of appropriation (Karsenti & Bugmann, 2019).

#### An emerging preference towards robotics unplugged activities

Section 4.1.2 and Fig. 2 show that although the proportion of adopters decreased in the second year, probably also partly due to COVID-19 interrupting the school year, during Year 2 the proportion of teachers adopting RU activities in their classrooms was greater than the one of teachers adopting CSU activities, in contrast with the results of Year 1. The exploration of the motivations beyond this fact is of fundamental importance for understanding the role that ER can play in formal education. As an example, a teacher commented that contrarily to what most expect, the RU activities require less material preparation than the CSU counterparts, and are easier to transpose from the training sessions to the classrooms. At the same time, the RU activities are generally considered as more technical, more complex to handle in a classroom, and requiring more teaching time than the CSU counterparts. Indeed, the results provide concrete testimony of the extent to which time can be a prominent barrier to ER adoption, as already brought up by other studies (Kradolfer et al., 2014; Chevalier et al., 2016; Mondada et al., 2017; Castro et al., 2018; Negrini, 2020) and the need for a community and support within the establishments (El-Hamamsy et al., 2020). Having dedicated time in the curriculum seems all the more critical to ensure that teachers continue to integrate ER in their practices without impacting the rest of the curriculum, a concern often raised by teachers.

The global preference towards unplugged content, robotics based or not, highlights the importance of designing developmentally appropriate ER tools (Elkin et al., 2018) with multiple interaction means (El-Hamamsy et al., 2020). Whilst one could question whether unplugged activities are as efficient as the plugged alternatives in terms of learning outcomes, there is an increasing number of studies investigating the question. In particular, del Olmo-Muñoz et al. (2020) showed that “unplugged activities improve computational thinking skills in early Primary Education” and are beneficial when it comes to motivation and gender issues. Indeed, our findings confirm the emerging hypothesis that the success of ER activities in formal education relies on moving the focus away from “the robot” to consider the broader Educational Robotics System (i.e., the tasks, the interface and the robot; Giang et al., 2019), to be put in relation with the instruction modality, the learning objectives and the assessment tools and goals (Giang, 2020).

#### Favouring one type of activity is not indicative of a lack of seriousness

The adoption analysis reported in Section 4.1.3 seeks to determine how seriously teachers adopted the proposed activities and whether this is correlated with a preference towards one type of activity. Since two of the three dimensions composing adoption seriousness are capped by the number of activities (namely, quantity and completion), and being this number quite small for RU activities, the seriousness metric for RU activities mainly relies on the frequency dimension. Conversely, given the larger number of CSU activities proposed during the CS-CPD program, the seriousness metric for CSU activities is balanced over all three dimensions. Relative grading, which would not by impacted by a normalisation on the number of activities per activity type, should nonetheless ensure the comparability between the two metrics. Moreover, biases in the construction of the metric would have a similar impact on the two years of analysis, thus allowing for their comparison.

As Fig. 3 shows, RU activities are generally adopted to similar extents as the CSU activities. Whilst most teachers adopt both types of activities, some seem to favour one type over the other (see Fig. 2), with an increasing shift towards robotics activities. The expression of such a preference does not seem to impact the teacher’s seriousness about the CS curriculum as a whole (see Fig. 3). Indeed, there are “serious” teachers that favour one type over the other and “non serious” teachers that adopt both types of activities lightly. This demonstrates the importance of including both types of activities, which rely on different instruction modalities to cover similar concepts. We hypothesise that this diversity contributes to an increase in the proportion of teachers adopting the proposed activities durably in their classrooms.

### On the perception of ER and its relation to adoption

#### K-4 non-voluntary teachers attain a positive perception of ER through the CS-CPD program

The analysis reported in Section 4.2.1 and Fig. 4 suggests that teachers following the CS-CPD globally possess a positive view of ER. Whilst several studies have found similar results (Chevalier et al., 2016; Castro et al., 2018), the notable element here is that our study involved teachers participating in a mandatory CS-CPD program, and thus not only voluntary, self-motivated participants. Our analysis can thus contribute to assess the validity, at a broad scale, of hypotheses and findings so far typically confined to smaller-scale initiatives.

#### Bridging the gap between novices and pioneers in perception and adoption of ER activities

As detailed in Sections 4.2.2 and 4.2.3 (see Tables 6 and 7), we focus on a teacher’s willingness to recommend ER to others and their prior experience with ER as starting points to understand the interplay between perception and adoption, and the factors influencing both. On the one hand, recommendation appears to be significantly correlated with both perception and adoption, in line with the fact that recommendation is often highly correlated with a customer’s return in the literature on customer satisfaction (Danaher & Haddrell, 1996). On the other hand, prior experience with ER seems to have a significant impact on a teacher’s self-efficacy, confirming the importance of prior experience on self-efficacy (Prieto & Altmaier, 1994) in the case of ER, but not on adoption, demonstrating that adoption is complex (Straub, 2009) and that there are more factors at play when it comes to determining whether a teacher will adopt any type of technological innovation or not (King & He, 2006). Surprisingly (and comfortingly) adoption was found to be uncorrelated with age, gender, teaching experience and even prior experience with ER, proving that ER can be successfully introduced into formal education without being constrained to a particular group of people through adequate professional development which can support equity in the introduction of CS and ER into formal education. The CS-CPD program considered in our study therefore appears to have successfully peaked novice teachers’ interest in robotics, leading them to successfully integrate robotics into their practices, as much as ER pioneer teachers would. The results support the findings of Chevalier et al., (2016) who highlighted the importance of engaging in ER activities so teachers may gain in confidence and therefore break some of the stereotypes around ER, notably in terms of usability.

Although the results are globally positive and support the idea to introduce ER within a CS curriculum, there is still a lack of understanding of the factors influencing teachers’ perception of robotics and, more importantly, their decision to adopt it or not in their practices. To this aim, we are currently investigating the link between teacher profiles and their perception and adoption of ER activities.

## Conclusion

Despite the well-known benefits that Educational Robotics (ER) has to offer, to this day it is mostly confined to formal settings and voluntary initiatives. In this article we analyse the results of a Continuing Professional Development (CPD) program for primary school teachers which, while focusing on concepts of Computer Science (CS) and Digital Education at large, integrated Educational Robotics as a versatile tool to teach CS. Inspired by the principles of translational research, our analyses aim to provide useful insights both for the practitioners in the field (teachers, professionals in charge of the CPD and education officers) and researchers investigating educational reforms or educational technologies. From the former perspective, our objective was twofold: (1) to determine whether CS curricular reforms (currently ongoing or being discussed in a large and growing number of countries) are a viable avenue for the introduction of ER activities in formal education, and (2) to assess the extent to which CS as a discipline can benefit from the additional ER activities, through the evaluation of teacher practices. From the latter perspective, we: (1) explored the tangled relationship between perception, adoption and recommendation, all the more interesting in the context of a mandatory CPD program, and (2) investigated factors influencing adoption, specifically looking at the relevance of prior experience with ER.

The CPD program was piloted with all the teachers (approx. 350) employed in 10 primary schools of the region, chosen as a representative sample of the whole region. The results suggest that the presence of robot-based ER activities alongside robot-less CS activities was beneficial for the CS program, allowing teachers to pick the type best aligned with their preferences and integrate it in their practice to teach CS concepts. Quite interestingly, the adoption of ER activities increases from the first to the second year, with RU activities even surpassing CSU activities in terms of number of hours devoted to them in classrooms, as well as the overall proportion of teachers adopting them. This finding suggests that ER can indeed benefit from the association with CS to enter formal education. The analysis of teachers’ perception of ER revealed that participants had a globally positive perception of ER and its benefits, a finding supported by the high adoption rates registered throughout the two-year study. Comfortingly, adoption was found to be uncorrelated with age, gender and even prior experience with ER. We hope that this result will contribute to removing stereotypes and barriers still impacting the public’s opinion of the discipline.

Finally, to successfully introduce ER into teacher practices, the robotics community in particular must engage in discussions around curricular reform, and offer new ER situations that help further student learning. This can be achieved by proposing adequate and developmentally appropriate tools elaborated based on specific guidelines (Giang et al., 2019), with the curriculum, learning objectives and assessment methods in mind (Giang, 2020), and ideally in co-construction with teachers. Finally, all development can be piloted with voluntary teachers, as the results are likely to generalise to teachers who have followed an ER-CPD based on the CS and Robotics Integration Model (El-Hamamsy et al., 2020).

## Data Availability

The data is accessible at 10.5281/zenodo.4081555.

## Footnotes

1 Involving the use of the BlueBot and Thymio II robots.

2 Extrinsic motivation, is closely related to the notion of controlled motivation in self-determination theory, and “concerns behaviors done for reasons other than their inherent satisfactions” (Ryan & Deci, 2000), they are based on external sources with the objective of attaining external rewards or avoiding punishment. Individuals thus feel pressured to do things, rather than be autonomously be motivated to do them.
